# Temporal interference stimulation for treating Wilson disease: A prospective clinical and functional MRI study

**DOI:** 10.1016/j.neurot.2026.e01097

**Published:** 2026-10-12

**Authors:** Nan Cheng, Yanming Wang, Huiyu Du, Guofang Yu, Xiaojing Han, Yangfei Huang, Chunsheng Xu, Bensheng Qiu, Ting Dong

**Affiliations:** a Department of Neurology, The First Affiliated Hospital of Anhui University of Chinese Medicine, Hefei, Anhui, 230031, China; b Medical Imaging Center, Department of Electronic Engineering and Information Science, University of Science and Technology of China, Hefei, Anhui 230026, China

**Keywords:** Wilson disease, Temporal interference stimulation, Putamen, Resting-state functional MRI, Neuromodulation

## Abstract

Wilson disease (WD) is an inherited disorder of copper metabolism caused by ATP7B mutations, and persistent neurological deficits, especially dystonia and motor disability, may remain despite standard anti-copper therapy. The putamen is a deep gray-matter nucleus frequently affected by copper deposition in WD and may therefore represent a plausible target for non-invasive neuromodulation. This prospective, single-arm, open-label study evaluated the clinical efficacy and neural mechanisms of right putamen-targeted temporal interference stimulation (TIS) in patients with WD. Twenty-five patients received right putamen-targeted TIS once daily for 10 sessions. Clinical outcomes were assessed using the Unified Wilson's Disease Rating Scale (UWDRS) total score and its neurological, hepatic, and psychiatric subscale scores. Resting-state functional magnetic resonance imaging (rsfMRI) was used to evaluate regional homogeneity (ReHo) and seed-based functional connectivity (FC) of the right putamen. Paired tests compared pre- and post-treatment changes, and Spearman correlation analysis examined associations between imaging changes and clinical improvement. After treatment, the UWDRS total score decreased significantly from 59.40 ± 30.54 to 44.12 ± 24.96, with concurrent improvements in neurological, hepatic, and psychiatric subscale scores. The rsfMRI analysis showed increased ReHo in the right putamen and enhanced FC between the right putamen and right Rolandic operculum. After correction for multiple comparisons, greater increases in right putamen ReHo correlated significantly with larger reductions in UWDRS total and neurological scores. In this exploratory study, these preliminary findings suggest that right putamen-targeted TIS may improve clinical symptoms in WD, potentially through enhanced local functional synchronization within the putamen.

## Introduction

Wilson disease (WD) is an autosomal recessive disorder of copper metabolism caused by pathogenic variants in the ATP7B gene [1]. These mutations impair biliary copper excretion and lead to pathological copper accumulation predominantly in the liver and brain [2]. Although the global prevalence of WD has traditionally been estimated at approximately 1 in 30,000 individuals, recent genetic and regional epidemiological studies suggest that the actual disease burden may be higher than conventional clinical estimates [3]. This issue is particularly relevant in China, where a large population base and regional enrichment of common ATP7B variants may translate a conventionally rare disorder into a considerable number of affected patients [4]. Notably, WD is among the few inherited neurodegenerative disorders for which disease-modifying therapies are available. Nevertheless, neurological involvement remains a major determinant of long-term disability [5]. A substantial proportion of patients develop neurological manifestations, including dystonia, parkinsonism, dysarthria, tremor, gait disturbance, and neuropsychiatric symptoms, which are closely associated with copper-related injury of deep gray matter structures [6]. Standard pharmacotherapy with copper chelators and zinc salts is essential for reducing systemic copper overload and improving hepatic outcomes; however, their ability to reverse established neurological deficits remains limited [7]. Early neurological deterioration after the initiation of anti-copper therapy has also been reported, particularly in patients with WD. These observations suggest that, once brain injury has been established, copper reduction alone may be insufficient to fully restore neurological function [8], [9]. Adjunctive interventions targeting disease-relevant brain circuits may therefore warrant investigation for patients with persistent neurological manifestations.

The limitations of conventional medical therapy have motivated increasing interest in neuromodulation-based approaches for neurological WD. Repetitive transcranial magnetic stimulation (rTMS), a representative non-invasive brain stimulation technique, has been explored in movement disorders through its effects on cortical excitability and motor-related brain function [10], [11]. In WD, previous studies have investigated rTMS for hand dystonia and upper-limb motor dysfunction, with preliminary evidence suggesting potential symptomatic benefit when stimulation is applied over the primary motor cortex [12]. Transcranial electrical stimulation (tES), including transcranial direct current stimulation (tDCS) and transcranial alternating current stimulation (tACS), has been explored in dystonia and other movement disorders; however, clinical evidence for tES in WD remains scarce, and no dedicated clinical evidence for tACS in WD has yet been established [13], [14]. Nevertheless, the anatomical reach of these conventional non-invasive stimulation approaches remains limited. Their primary effects are concentrated in superficial cortical regions, whereas direct and focal modulation of deep subcortical nuclei remains technically challenging because of the depth-focality trade-off inherent to non-invasive stimulation [15]. This limitation is especially relevant for WD, as neurological manifestations are closely associated with abnormalities in deep gray matter nuclei, particularly the basal ganglia [16]. Therefore, non-invasive approaches capable of more effectively reaching deep brain targets may be more suitable for exploring neuromodulation strategies in WD.

Temporal interference stimulation (TIS) has recently emerged as a promising method for non-invasive deep brain neuromodulation [15]. By delivering two or more high-frequency electric fields with slightly different frequencies, TIS can generate a low-frequency envelope at the intersection of the fields, thereby enabling modulation of neural activity within deep brain regions while reducing direct stimulation of overlying cortical tissue [17]. The original proof-of-concept study demonstrated that temporally interfering electric fields could non-invasively stimulate deep hippocampal neurons in mice without recruiting the overlying cortex [18]. Subsequent human studies further suggested that TIS can modulate hippocampal activity and episodic memory [41], as well as influence striatal activity and motor skill learning [19]. A recent systematic review of human TIS applications reported target engagement in the motor cortex, basal ganglia, and hippocampus, with early clinical exploration in Parkinson's disease, essential tremor, epilepsy, and depression-related protocols [20]. Although the clinical application of TIS remains at an early stage, these findings support further investigation of TIS as a non-invasive approach for modulating deep brain structures.

For WD, the putamen represents a neuroanatomically plausible and clinically relevant target for TIS. As a major component of the lenticular nucleus and basal ganglia, the putamen is frequently involved in WD-related copper deposition [21], MRI signal abnormalities, atrophy, and functional alterations. MRI studies of WD have shown that the putamen is among the most commonly affected regions, with one clinical MRI study reporting putaminal involvement in 85.3% of patients [22]. Resting-state functional magnetic resonance imaging (rsfMRI) evidence further indicates that abnormalities of the lenticular nucleus, including putaminal and pallidal subregions, are associated with dystonia and other clinical manifestations in WD [23], [25]. Multiparametric MRI studies also suggest that microstructural and susceptibility changes in deep gray matter nuclei correlate with neurological severity [24]. Functionally, the putamen is involved in motor planning, motor execution, movement coordination, and muscle tone regulation, all of which are relevant to the motor manifestations of WD [16], [25]. Consequently, this study targeted the putamen to evaluate the efficacy of TIS in modulating this deep brain region for WD treatment, utilizing both clinical and neuroimaging assessments.

The present study employed a prospective, single-arm, open-label interventional design to investigate putamen-targeted TIS in patients with WD. Clinical outcomes were assessed using the Unified Wilson's Disease Rating Scale (UWDRS) and its neurological, hepatic, and psychiatric domains, while rsfMRI was used to characterize changes in putamen-centered regional activity and FC. By combining standardized clinical assessment with functional neuroimaging, this study explored whether stimulation of a disease-relevant subcortical target is accompanied by changes in clinical manifestations and putamen-related brain function. These findings may provide a basis for further controlled studies of putamen-targeted neuromodulation as an adjunctive approach for WD.

## Methods

### Study design and ethical approval

This was a prospective, single-arm, open-label exploratory interventional study with a pre-post treatment design. The study was conducted at the First Affiliated Hospital of Anhui University of Chinese Medicine. The study protocol was reviewed and approved by the Medical Ethics Committee of the First Affiliated Hospital of Anhui University of Chinese Medicine under approval number 2026AH-20-01 and 2026AH-144. All study procedures were performed in accordance with the approved protocol, the ethical principles of the Declaration of Helsinki, and applicable institutional requirements [26].

Before enrollment, all participants were informed of the study objectives, intervention procedures, potential benefits, and possible risks. Written informed consent was obtained from all participants prior to participation. Participant confidentiality was protected throughout the study, and clinical and imaging data were anonymized before analysis.

### Participants and eligibility criteria

Patients were recruited from the outpatient and inpatient services of the First Affiliated Hospital of Anhui University of Chinese Medicine. Eligible participants were required to meet the following criteria: age between 18 and 45 years; a confirmed diagnosis of WD; dystonia as a clinically relevant neurological manifestation; ability to complete the scheduled clinical assessments; willingness to provide written informed consent; and an ability to comply with the intervention and follow-up procedures.

The exclusion criteria were as follows: age younger than 18 years or older than 45 years; a personal history of epileptic seizures or a family history of epilepsy; previous cranial surgery; intracranial or cardiac metallic implants; contraindications to magnetic resonance imaging; scalp injury or other local conditions precluding transcranial stimulation; use within the preceding 12 weeks of medications specifically intended to affect dystonia; other neurological or systemic disorders that could produce dystonia; marked psychiatric abnormalities interfering with study participation; severe limb tremor or deformity that precluded reliable tone-related assessment; pregnancy; refusal to provide informed consent; and poor expected adherence.

Prespecified withdrawal criteria included intercurrent illness that rendered continued participation inappropriate, substantial non-adherence to the study protocol, and voluntary withdrawal from treatment or follow-up.

### Temporal interference stimulation

All participants received right putamen-targeted TIS using a four-channel temporal interference stimulation device (FQ-WGD0423, Fuqing Medical Co., Ltd., Hefei, Anhui, China). The stimulation protocol delivered two high-frequency sinusoidal alternating currents with carrier frequencies of 1300 and 1430 Hz, producing a 130-Hz envelope modulation. This 130-Hz envelope frequency was selected based on established high-frequency basal ganglia neuromodulation paradigms and prior TIS studies targeting motor circuits [27]. Electrode placement was guided by the international 10-10 EE G system. Individualized electrode montages and current allocations were determined via computational optimization to maximize the interferential envelope at the right putamen, while maintaining the peak current at each electrode below 2.5 mA and the total injected current below 10 mA [28], [29]. Detailed participant-specific electrode configurations, current allocations, and simulated target electric-field values are provided in Supplementary Table S1; a representative simulation of the interferential envelope distribution is shown in Supplementary Fig. S1 to illustrate target focality. Each session comprised a 30-s ramp-up period, 30 min of continuous stimulation, and a 30-s ramp-down period. Stimulation was administered once daily across two 5-day blocks separated by a 2-day rest period, totaling 10 sessions per participant. Throughout each session, participants remained comfortably seated, awake, and at rest, without performing concurrent behavioral tasks, motor training, or additional rehabilitation interventions. Adverse sensations and events-including headache, dizziness, scalp tingling, and skin discomfort-were systematically monitored and recorded throughout the treatment course.

### Clinical outcomes and scale assessment

Clinical outcomes were assessed at baseline and after the intervention using standardized clinical rating scales. The primary clinical outcome was the change in the UWDRS total score, which was used to evaluate overall disease severity in patients with WD [30]. Secondary clinical outcomes included changes in the neurological function score, hepatic function score, and psychiatric symptom score, which were used to assess domain-specific clinical manifestations. All assessments were performed according to a standardized evaluation procedure. Higher scores indicated more severe clinical impairment. Post-treatment scores were compared with baseline scores to evaluate intervention-related clinical changes.

### MRI acquisition and resting-state fMRI preprocessing

Magnetic resonance imaging was performed before and after the intervention using a 3.0-T GE DISCOVERY MR750 scanner equipped with an 8-channel head coil. The imaging protocol included a conventional localizer, high-resolution three-dimensional T1-weighted BRAVO imaging, and rsfMRI. T1-weighted images were acquired using a 3D BRAVO sequence with the following parameters: repetition time = 8.16 ms, echo time = 3.18 ms, flip angle = 12°, matrix size = 256 × 256, field of view = 256 × 256 mm^2^, voxel size = 1 × 1 × 1 mm^3^, and slice thickness = 1 mm. The rsfMRI data were acquired using a gradient-echo single-shot echo-planar imaging sequence with the following parameters: repetition time = 2000 ms, echo time = 30 ms, flip angle = 90°, matrix size = 64 × 64, field of view = 220 × 220 mm^2^, in-plane resolution = 3.434 × 3.434 mm^2^, slice thickness = 3 mm. During resting-state scanning, participants were instructed to remain still with their eyes closed, stay awake, and avoid systematic thinking.

The rsfMRI data were preprocessed using a standardized pipeline applied identically to baseline and post-intervention scans. The first 10 vol were discarded, followed by slice-timing correction, head-motion correction, spatial normalization to Montreal Neurological Institute space, resampling to 3 × 3 × 3 mm^3^ isotropic voxels, linear detrending, and temporal band-pass filtering at 0.01–0.08 Hz. Participants were excluded from MRI-based analyses if head motion exceeded 3.0 mm in translation or 3.0° in rotation in any direction. MRI analyses were performed only in participants with complete paired pre- and post-intervention MRI data and acceptable image quality after preprocessing.

### Right putamen regional activity analysis based on ReHo

As the prespecified stimulation target, the right putamen was also defined as the primary region of interest for analyzing local brain activity. For standardized and reproducible MRI quantification across participants and time points, the right putamen region of interest used for signal extraction was defined in Montreal Neurological Institute space using the Automated Anatomical Labeling atlas with 116 regions, hereafter referred to as AAL116 [31]. Regional homogeneity (ReHo) was used to assess local synchronization of spontaneous resting-state brain activity within the right putamen. ReHo quantifies the similarity between the time series of a given voxel and those of its neighboring voxels using Kendall's coefficient of concordance. Voxel-wise ReHo maps were calculated using a 27-voxel neighborhood, including each voxel and its 26 nearest neighboring voxels. ReHo calculation was performed after realignment, spatial normalization, linear detrending, and temporal band-pass filtering at 0.01–0.08 Hz. To reduce global interindividual and interscan variability, ReHo maps were normalized by the whole-brain mean ReHo value and then spatially smoothed using a Gaussian kernel of 6 mm full width at half maximum.

For each participant, the mean ReHo value within the AAL116-defined right putamen region of interest was extracted before and after the intervention. The change in right putamen ReHo was calculated as the post-intervention value minus the baseline value.

### Right putamen functional connectivity analysis

ReHo reflects local functional synchronization within the stimulation target but does not capture functional communication between the target and other brain regions. Therefore, seed-based functional connectivity (FC) analysis was performed using the right putamen as the seed region. The mean resting-state time series of the right putamen was extracted for each participant. Pearson correlation coefficients were calculated between the right putamen time series and the mean time series of each predefined brain region. The predefined brain regions were derived from the AAL116 atlas, which provided 116 anatomical regions for region-level FC analysis.

Correlation coefficients were transformed into Fisher z values before statistical analysis. For each region, FC change was calculated as the post-intervention z value minus the baseline z value. Region-level FC changes were assessed across predefined brain regions. False discovery rate (FDR) correction was applied to account for multiple comparisons.

Neuroimaging preprocessing and quantitative analyses were performed in MATLAB R2025b using Statistical Parametric Mapping version 12 and DPABI version 26. ReHo and FC measures were derived within the same MATLAB-based workflow, and brain visualizations were generated using BrainNet Viewer.

### Statistical analysis

Continuous variables were summarized as mean ± standard deviation, and categorical variables were summarized as counts and percentages. Within-subject changes were calculated as post-intervention values minus baseline values. Pre-post differences in clinical and neuroimaging measures were assessed using paired t tests. FC coefficients were transformed into Fisher z values before group-level statistical testing. Region-level right putamen-based FC analyses were corrected for multiple comparisons using the Benjamini-Hochberg FDR procedure. All tests were two-tailed, and P < 0.05 were considered statistically significant.

### Imaging-clinical association analysis

Imaging-clinical association analyses were performed in the MRI-complete subset to explore whether intervention-related neuroimaging changes were associated with clinical improvement. Imaging measures included changes in right putamen ReHo and changes in right putamen-based FC in regions showing significant pre-post alterations. Clinical measures included changes in UWDRS total score and domain scores, calculated as post-intervention values minus baseline values. Spearman correlation analysis was used for these exploratory associations, and FDR correction was applied where appropriate for multiple correlation tests.

### Data sharing statement

The data that support the findings of this study are not publicly available because of ethical and privacy restrictions. De-identified data may be made available from the corresponding authors upon reasonable request and after approval by the relevant ethics committee and institutional authorities.

## Results

### Baseline characteristics and clinical outcomes

A total of 25 patients with WD were included in the clinical analysis. The cohort was predominantly male, with 20 male patients and 5 female patients. The mean age was 29.88 ± 8.02 years, and the mean body mass index was 21.25 ± 3.11 kg/m^2^. At baseline, the mean UWDRS score was 59.40 ± 30.54. The baseline characteristics of the cohort are summarized in Table 1 (see Fig. 1).

**Table 1:** Baseline characteristics of the study cohort.

Characteristic	Overall cohort (n = 25)
Sex	
Male, n (%)	20 (80.00)
Female, n (%)	5 (20.00)
Age, years	29.88 ± 8.02
BMI, kg/m^2^	21.25 ± 3.11
UWDRS score	59.40 ± 30.54

Data are presented as mean ± standard deviation or n (%), unless otherwise indicated. UWDRS = Unified Wilson Disease Rating Scale; BMI = body mass index.

Following right putamen-targeted TIS, reductions were observed in the UWDRS total score and domain-specific scores. The UWDRS total score decreased significantly from 59.40 ± 30.54 to 44.12 ± 24.96, with a mean change of −15.28 points (95% CI, −19.70 to −10.86; Cohen's d_z_ = −1.43; t = −7.139, P < 0.001). Significant reductions were also observed in the neurological function score, from 47.24 ± 27.55 to 35.28 ± 22.20, with a mean change of −11.96 points (95% CI, −15.73 to −8.19; Cohen's d_z_ = −1.31; t = −6.549, P < 0.001); hepatic function score, from 4.24 ± 2.17 to 3.20 ± 1.98, with a mean change of −1.04 points (95% CI, −1.68 to −0.40; Cohen's d_z_ = −0.68; t = −3.375, P = 0.004); and psychiatric symptom score, from 8.00 ± 4.67 to 5.64 ± 3.50, with a mean change of −2.36 points (95% CI, −3.42 to −1.30; Cohen's d_z_ = −0.91; t = −4.574, P < 0.001). These paired clinical changes are shown in Fig. 2 and summarized in Table 2.

**Fig. 1:**
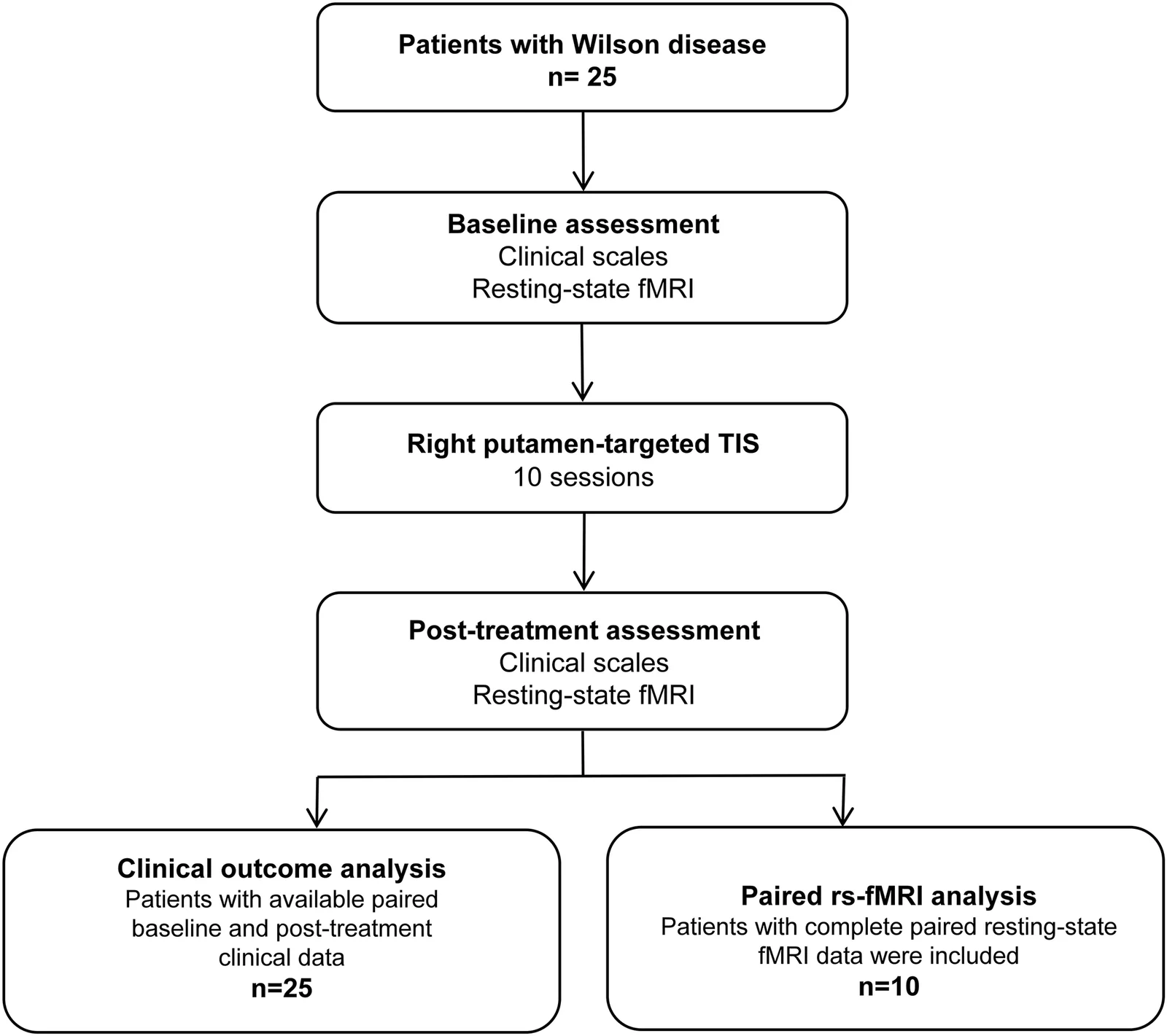
**Study flow and analysis cohorts.** Twenty-five patients with Wilson disease underwent baseline clinical assessment and resting-state fMRI, followed by one treatment course of right putamen-targeted temporal interference stimulation consisting of 10 sessions. Post-treatment clinical and resting-state fMRI assessments were then performed. All 25 patients had paired clinical data and were included in the clinical outcome analysis. Ten patients had useable paired resting-state fMRI data and were included in the imaging analysis, whereas 15 patients were excluded from paired resting-state fMRI analysis because of incomplete paired imaging data or imaging-quality limitations.

**Fig. 2:**
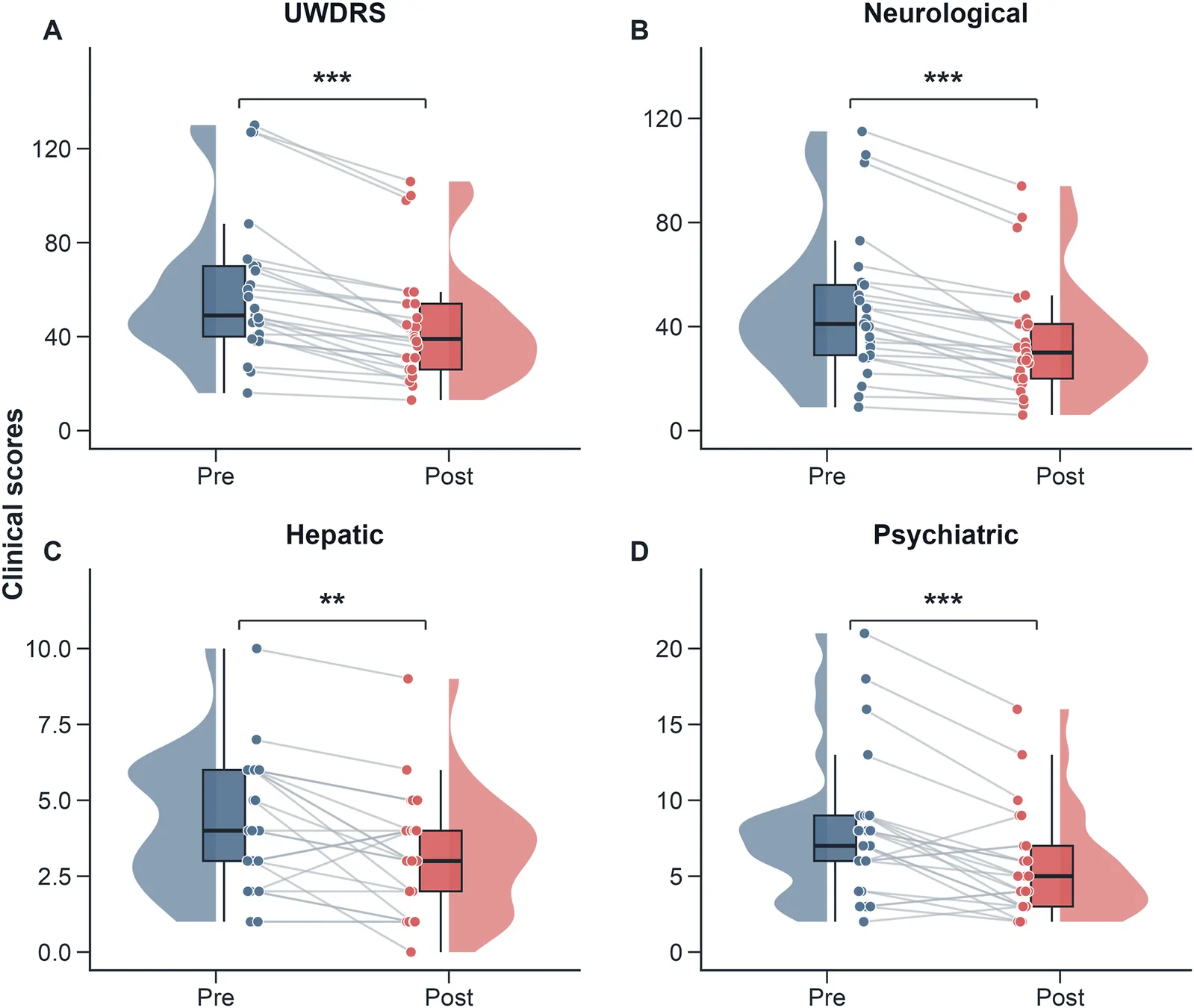
**Clinical score changes after right putamen-targeted temporal interference stimulation.** Clinical outcomes were compared between baseline and post-treatment assessments in patients with Wilson disease. Panels A–D show changes in the Unified Wilson Disease Rating Scale (UWDRS) total score, neurological score, hepatic score, and psychiatric score, respectively. Each point represents an individual patient (n = 25), and gray lines connect paired baseline and post-treatment measurements from the same patient, illustrating individual trajectories of clinical change. Boxplots and density distributions summarize the overall score distributions before and after intervention. Lower post-treatment scores indicate clinical improvement. UWDRS, Unified Wilson Disease Rating Scale.

**Table 2:** Pre- and post-treatment comparisons of clinical scores.

Outcome	Pre-treatment	Post-treatment	Mean change (95% CI)	Cohen's d_z_	t value	*P* Value
UWDRS total	59.40 ± 30.54	44.12 ± 24.96	−15.28 (−19.70, −10.86)	−1.43	−7.14	<0.001
Neurological	47.24 ± 27.55	35.28 ± 22.20	−11.96 (−15.73, −8.19)	−1.31	−6.55	<0.001
Hepatic	4.24 ± 2.17	3.20 ± 1.98	−1.04 (−1.68, −0.40)	−0.68	−3.38	0.004
Psychiatric	8.00 ± 4.67	5.64 ± 3.50	−2.36 (−3.42, −1.30)	−0.91	−4.57	<0.001

Values are presented as mean ± SD. Mean change was calculated as Post-treatment minus Pre-treatment values; negative values indicate a reduction in UWDRS scores, representing clinical improvement. Cohen's d_z_ was calculated based on paired differences, with negative values indicating a decrease after intervention. Paired t-tests were used to compare pre- and post-treatment outcomes.

### Right putamen ReHo and functional connectivity changes

Among the clinical cohort, paired pre- and post-intervention MRI data were available for 10 participants. During preprocessing, one participant was excluded because the maximum head motion exceeded the prespecified quality-control threshold (>3.0 mm translation or >3.0° rotation). Therefore, nine participants were included in the final ReHo, FC, and imaging-clinical association analyses. After establishing the clinical changes, the analysis next examined whether right putamen-targeted stimulation was accompanied by alterations in rsfMRI measures. The right putamen was first assessed as the predefined stimulation target using ReHo. Across the paired imaging data, right putamen ReHo increased after intervention within the predefined target region. These changes werelocalized within the predefined right putamen region of interest and were visualized across axial, coronal, and sagittal sections in Fig. 3.

**Fig. 3:**
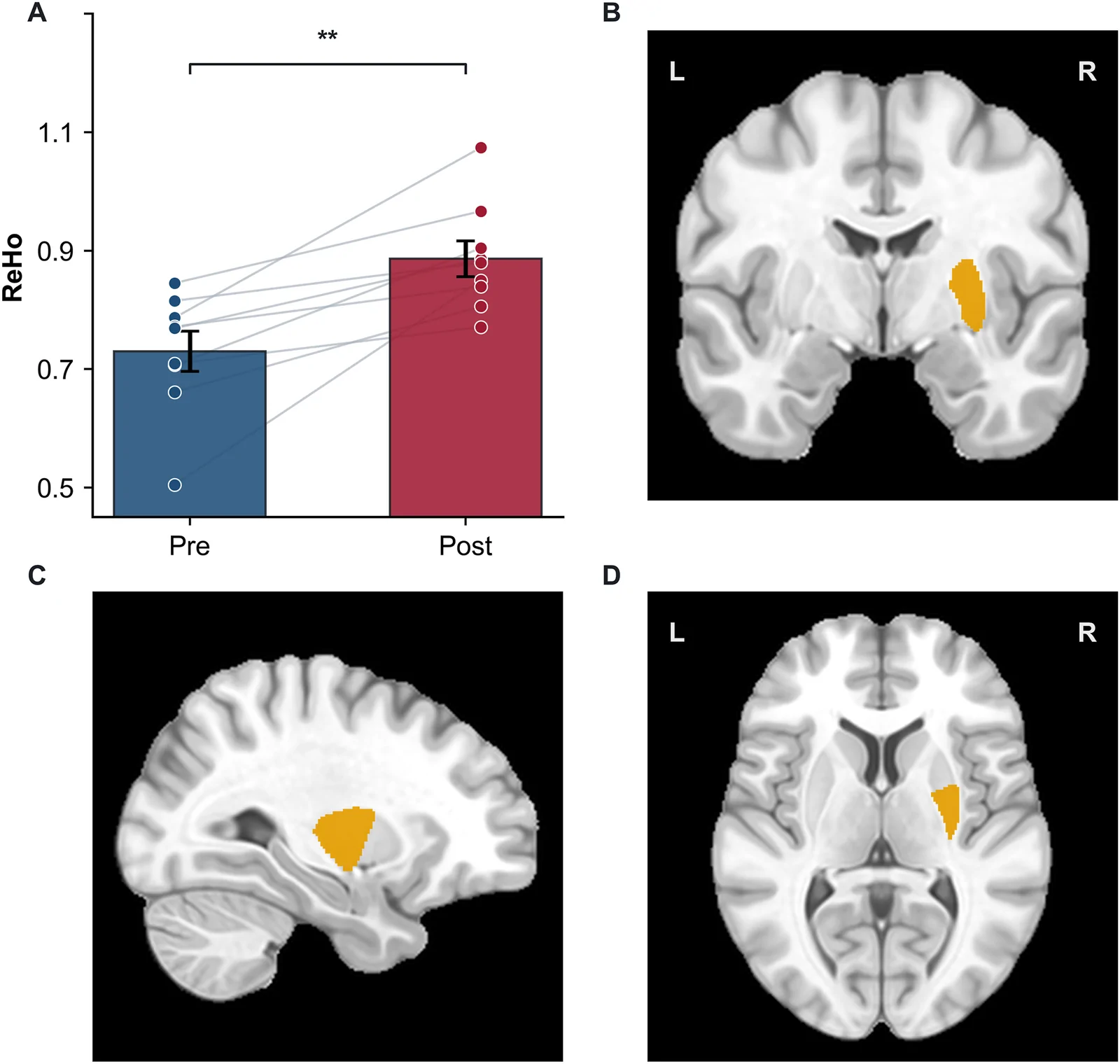
**Changes in right putamen ReHo after right putamen-targeted temporal interference stimulation.** Panel A shows paired right putamen ReHo values before and after right putamen-targeted temporal interference stimulation. Individual paired measurements are connected by gray lines, and bars summarize group-level values with error bars. Right putamen ReHo increased significantly after treatment. Panels B–D show the spatial distribution of treatment-related ReHo changes within the right putamen in coronal, sagittal, and axial views, respectively. ReHo changes were calculated as post-treatment values minus baseline values. Increased ReHo indicates enhanced local synchronization of spontaneous resting-state activity within the stimulation target. ReHo, regional homogeneity.

To further characterize putamen-related functional changes beyond local activity, seed-based FC analysis was performed using the right putamen as the seed region. After FDR correction, increased FC between the right putamen and the right rolandic operculum remained significant, with a post-pre Fisher z connectivity change of 0.326 and an FDR-corrected P value of 0.0478. The spatial distribution of the FDR-corrected connectivity change is shown in Fig. 4.

**Fig. 4:**
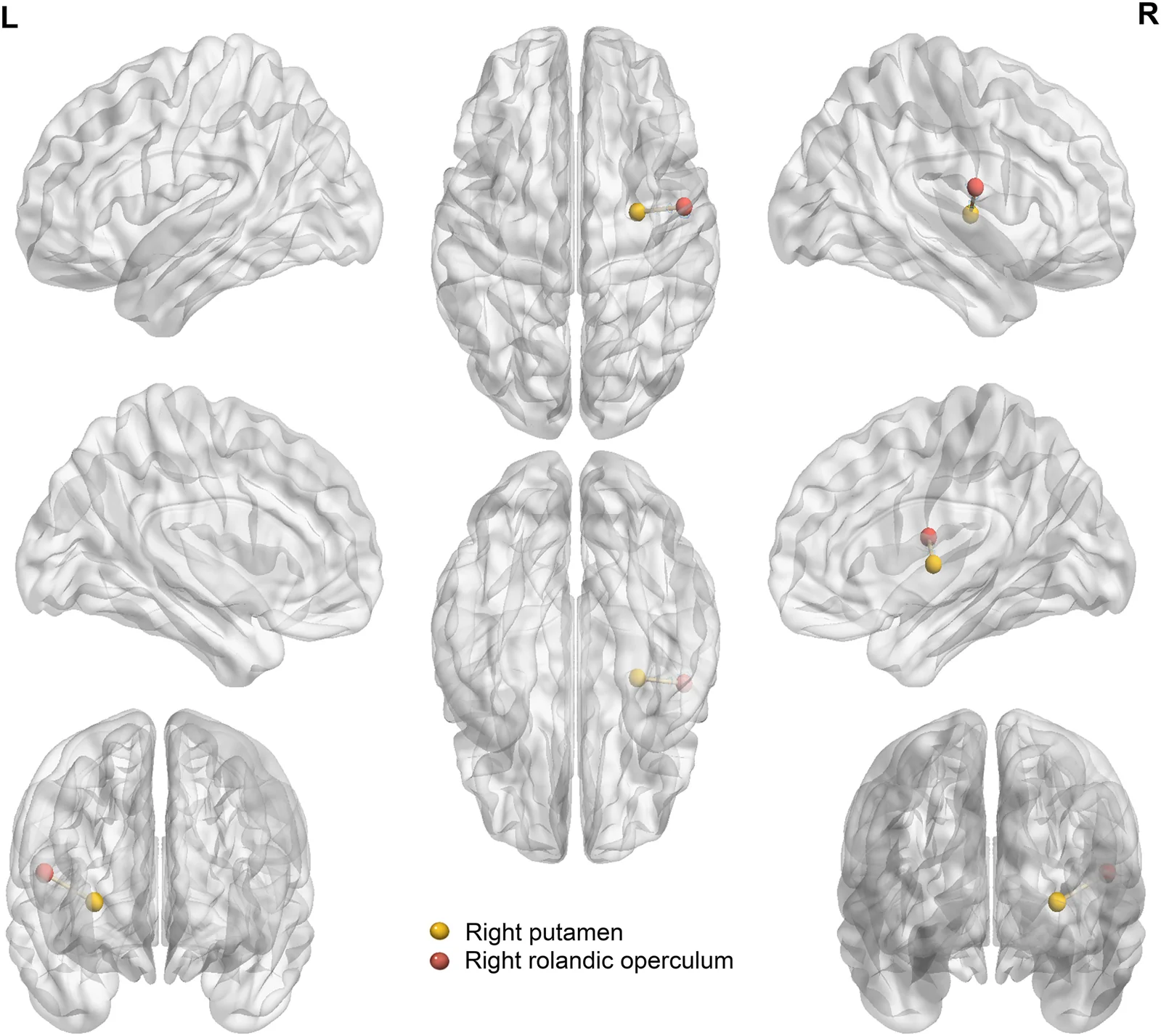
**Right putamen-right Rolandic operculum functional connectivity after stimulation.** Three-dimensional brain projections show the functional connectivity pattern between the right putamen seed and the right Rolandic operculum after right putamen-targeted temporal interference stimulation. The right putamen seed is shown as the deep subcortical node, and the right Rolandic operculum is shown as the cortical target node. The connecting line indicates increased seed-based functional connectivity between these regions. Multiple views are provided to illustrate the spatial relationship of the right putamen and right Rolandic operculum across lateral, medial, superior, inferior, anterior, and posterior perspectives. L, left hemisphere; R, right hemisphere; FC, functional connectivity.

### Imaging-clinical association

After identifying stimulation-related alterations in right putamen ReHo and putamen-centered FC, the analysis further assessed whether these neuroimaging changes were associated with clinical improvement. Greater increases in right putamen ReHo were associated with greater reductions in clinical scores. Specifically, the change in right putamen ReHo was negatively correlated with the change in UWDRS total score (rho = −0.828, P_FDR_ = 0.023). A stronger association was observed for the neurological function score (rho = −0.945, P_FDR_ < 0.001). In contrast, associations with hepatic function score and psychiatric symptom score did not survive FDR correction.

The relationship between right putamen-right rolandic operculum FC change and clinical score change was then examined. These correlations were weaker (rho = −0.243 for UWDRS total score; rho = −0.136 for neurological function score; rho = −0.341 for hepatic function score; rho = −0.414 for psychiatric symptom score) and did not reach statistical significance after correction (P_FDR_ > 0.05). These imaging-clinical association results are presented in Fig. 5.

**Fig. 5:**
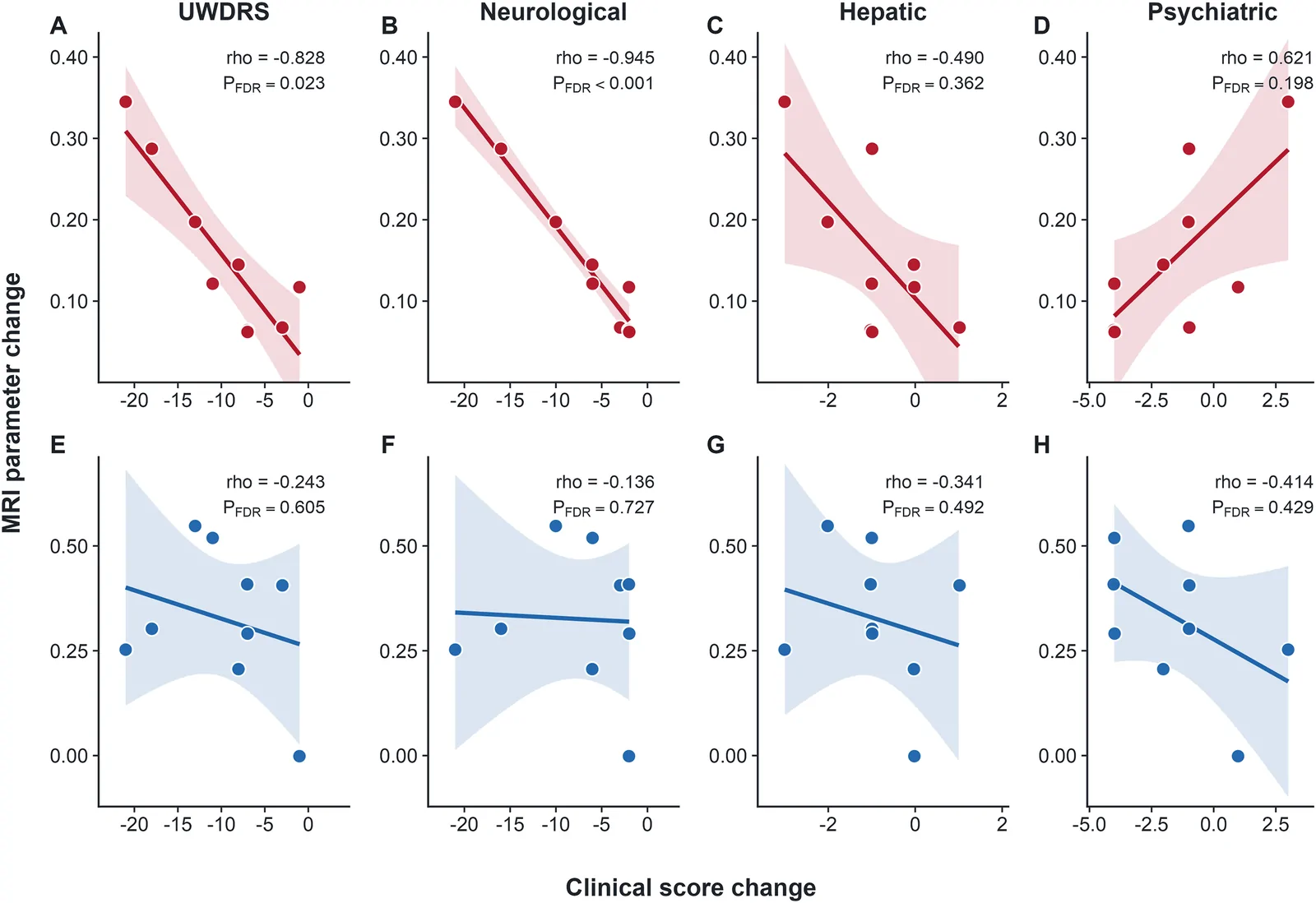
**Associations between imaging changes and clinical score changes.** Spearman correlation analyses were used to examine associations between treatment-related imaging changes and clinical score changes. Clinical score changes were calculated as post-treatment scores minus baseline scores; negative values therefore indicate clinical improvement. Imaging changes were calculated as post-treatment values minus baseline values. Panels A–D show associations between right putamen ReHo changes and changes in UWDRS total score, neurological function score, hepatic function score, and psychiatric symptom score, respectively. Panels E–H show associations between right putamen-right Rolandic operculum functional connectivity changes and the same clinical outcomes. Increased right putamen ReHo was significantly associated with greater reductions in UWDRS total score and neurological function score after FDR correction. Shaded areas represent confidence intervals around the fitted regression lines. FC, functional connectivity; FDR, false discovery rate; ReHo, regional homogeneity; UWDRS, Unified Wilson Disease Rating Scale.

### Treatment feasibility and safety

All 25 participants completed the scheduled TIS intervention consisting of 10 stimulation sessions. The stimulation protocol was generally well tolerated. Two participants experienced mild transient adverse events, including scalp tingling sensation (n = 1, 4.0%) and fatigue (n = 1, 4.0%). The scalp tingling sensation resolved spontaneously after brief rest, and the fatigue resolved after approximately 30 min of supine rest. No recurrence was observed during subsequent stimulation sessions or follow-up. No headache, dizziness, skin discomfort, nausea, or other stimulation-related adverse events were reported (Table S3).

## Discussion

The present study explored right putamen-targeted TIS in patients with WD, a rare inherited disorder in which prospective interventional neuroimaging studies are difficult to conduct. After one treatment course, patients showed reductions in the UWDRS total score and in the neurological, hepatic, and psychiatric domain scores, with the most prominent change observed in the neurological domain. In parallel, rsfMRI showed increased right putamen ReHo and enhanced FC between the right putamen and the right Rolandic operculum after correction for multiple comparisons. Imaging-clinical analysis further showed that the increase in right putamen ReHo was closely associated with improvement in both the total score and the neurological score. Taken together, these findings suggest that clinical changes were accompanied by alterations in local spontaneous activity within the predefined stimulation target. This pattern suggests that right putamen ReHo may serve as a neuroimaging correlate of clinical change after TIS; however, given the exploratory single-arm design, these findings should be interpreted as preliminary rather than definitive evidence of therapeutic efficacy.

The clinical findings should be considered in the context of the persistent treatment difficulty of neurological WD. Członkowska et al. emphasized that WD is one of the few treatable inherited neurodegenerative disorders, but also noted that neurological and psychiatric manifestations often contribute substantially to long-term disability [32]. Antos et al. reported that neurological deterioration may occur after anti-copper treatment, especially in patients who already present with neurological symptoms, suggesting that copper reduction does not always translate into rapid or complete neurological recovery [9]. Gao et al. and Yang et al. further highlighted that the true burden of WD may be underestimated, particularly in populations with enriched pathogenic ATP7B variants [33], [34]. These findings collectively highlight that neurological WD remains a clinically challenging phenotype, in which disease-modifying copper-lowering therapy may not fully address established neurological dysfunction. The observed reduction in clinical scores after TIS may therefore be viewed as an adjunctive clinical signal in a rare disease population with persistent neurological impairment. The finding also extends the work of Hao et al. who reported that high-frequency rTMS over the primary motor cortex improved upper-limb dystonia and Wilson disease-related clinical scores [35]. Unlike cortical stimulation, the present intervention targeted the putamen, a deep gray matter structure more closely aligned with the lesion distribution of neurological WD. Behavioral context may also influence responses to TIS. Ryan et al. reported that ventral-striatum TIS delivered during a defined behavioral intervention (breath-focused meditation) enhanced reward-related electrophysiological responses, supporting the potential relevance of behavioral state during stimulation [36]. In the present study, TIS was delivered during quiet rest without concurrent motor training or behavioral tasks. Given the role of the putamen in motor learning and sensorimotor integration, future studies should examine whether pairing putamen-targeted TIS with structured motor training could further enhance motor recovery and network plasticity in neurological WD.

The neuroimaging findings provide a functional context for the observed clinical changes. The putamen represents a biologically plausible target in WD because it is one of the deep gray matter structures most frequently affected by copper-related injury. Kalita et al. reported that putaminal abnormalities were present in a high proportion of patients with neurological WD [22]. Moreover, Yang et al. demonstrated that dysfunction of the lenticular nucleus, including putaminal and pallidal regions, was associated with dystonia and other clinical manifestations in WD [37]. These studies support the relevance of the putamen in a patient group characterized by dystonia and other motor symptoms. In the present study, increased right putamen ReHo after stimulation may reflect enhanced local temporal synchronization of spontaneous activity within the stimulation target. Since ReHo reflects local functional coherence rather than long-range coupling, it may be particularly suitable for detecting target-region changes after stimulation [31]. In addition to this local effect, seed-based FC analysis identified enhanced connectivity between the right putamen and the right Rolandic operculum after TIS, suggesting possible modulation of putamen-centered sensorimotor networks. The Rolandic operculum has been implicated in sensorimotor integration, particularly orofacial and speech-related motor control [38], [39]. This functional relevance is notable because dysarthria, dystonia, tremor, and orofacial motor impairment are common manifestations of neurological WD [40]. Therefore, the increased putamen–Rolandic operculum connectivity may reflect altered resting-state communication between a basal ganglia target and a sensorimotor-opercular region. Nevertheless, because resting-state FC does not directly index task-specific speech or motor performance, this network-level interpretation should be regarded as exploratory.

The imaging-clinical association further supports the potential relevance of target-region functional changes. Greater increases in right putamen ReHo were associated with larger reductions in both UWDRS total and neurological scores, suggesting that patients with larger increases in local putaminal synchronization tended to show greater reductions in clinical scores. This association was most pronounced for the neurological domain, which is consistent with the central role of the putamen in motor regulation and with the inclusion of patients with clinically evident dystonia [41]. Previous resting-state fMRI studies have demonstrated disrupted basal ganglia-thalamocortical circuits in WD, including abnormal local synchronization within the putamen and altered connectivity across broader brain networks [42]. Against this background, the observed association between increased right putamen ReHo and clinical improvement suggests that enhanced local functional coherence within this disease-relevant deep gray matter nucleus may serve as a plausible neuroimaging correlate of neurological response after TIS. This interpretation is consistent with emerging evidence that non-invasive TIS can influence activity in deep brain structures, including the striatum, and influence local neural activity and motor-related functions [43]. Collectively, these findings suggest that increased local synchronization within the right putamen may represent an imaging marker associated with clinical improvement after putamen-targeted TIS in WD.

Several limitations should be acknowledged in this study. First, although 25 patients were included in the clinical analysis, only 9 had complete paired MRI data meeting quality-control criteria. Accordingly, MRI-based longitudinal and imaging-clinical analyses were restricted to this subset. The reduced imaging sample reflects practical challenges in neurological WD, including movement symptoms, scanning intolerance, and head-motion artifacts that can limit acquisition of high-quality paired MRI data. Restriction to complete, quality-assured datasets strengthened the reliability of within-subject imaging comparisons but reduced statistical power and generalizability. Second, this was a prospective, single-arm, open-label study without randomization or sham stimulation. Although this design is suitable for an initial clinical and imaging exploration of a novel deep-targeted non-invasive intervention, randomized sham-controlled studies will be required to distinguish stimulation-specific effects from nonspecific treatment effects, placebo responses, and natural short-term fluctuations. Finally, the current analysis focused on short-term changes after one treatment course. Longer follow-up is needed to determine whether the observed clinical and imaging changes are sustained. Future randomized, double-blind, sham-controlled studies with larger samples are needed to establish the stimulation-specific effects and clinical effectiveness of putamen-targeted TIS in WD. The optimal envelope frequency also remains uncertain; although 130 Hz was selected based on prior basal ganglia neuromodulation and TIS studies [44], future studies should compare alternative frequencies to identify more effective stimulation parameters for WD.

In conclusion, this prospective interventional study provides preliminary evidence that right putamen-targeted TIS may be a feasible adjunctive neuromodulatory approach for patients with WD. After treatment, patients showed improvements in UWDRS total and domain-specific scores, accompanied by increased right putamen ReHo and enhanced FC between the right putamen and right Rolandic operculum. The association between increased right putamen ReHo and improvement in overall and neurological UWDRS scores further suggests that local functional changes within the stimulation target may be related to clinical response. Given the rarity of WD and the challenges of conducting interventional neuroimaging studies in this population, these findings should be interpreted as exploratory but encouraging. Future randomized, sham-controlled studies with larger samples and longer follow-up are warranted to determine the clinical efficacy, durability, and optimal stimulation parameters of right putamen-targeted TIS in neurological WD.

## Patient Consent

Not applicable.

## Ethical approval

The study protocol was reviewed and approved by the Medical Ethics Committee of the First Affiliated Hospital of Anhui University of Chinese Medicine under approval number 2026AH-20-01 and 2026AH-144.

## Data availability

Any supplementary data generated or analyzed during this study are available from the corresponding author upon reasonable request. We are committed to ensuring transparency and reproducibility of our research findings and will provide the necessary data to facilitate further investigation and validation by other researchers.

## Author contributions

Nan Cheng: study design, clinical execution, data collection, data analysis, and drafting of the manuscript. Yanming Wang: study design, functional MRI methodology, imaging analysis, and manuscript revision. Huiyu Du: functional MRI preprocessing, imaging analysis, and manuscript revision. Guofang Yu: clinical execution, data collection, and manuscript revision. Xiaojing Han: clinical assessment, data collection, and manuscript revision. Yangfei Huang: data organization, statistical analysis, and manuscript revision. Chunsheng Xu: clinical supervision, study execution, and manuscript revision. Bensheng Qiu: study supervision, functional MRI methodology, interpretation of imaging findings, and manuscript revision. Ting Dong: study conception, study design, project supervision, interpretation of results, and critical revision of the manuscript. All authors reviewed and approved the final version of the manuscript.

## Funding

This study was supported by the 10.13039/501100003995Anhui Provincial Natural Science Foundation (No. 2508085QH283), the Fundamental Research Funds for the 10.13039/100007231Central Universities (No. WK2100000048), the Anhui Provincial Chinese Medicine Tradition and Innovation Research Project Plan (No. 2025CCCX064), the Anhui University of Chinese Medicine 2025 Basic-Clinical Integration Special Program (No. JCLCA2025012), and the Scientific Research Project of the Anhui Provincial Department of Education (No. 2025AHGXZK50112).

## Declaration of competing interest

The authors declare that they have no financial disclosures or conflicts of interest relevant to the research covered in this manuscript.
