# Supplementary material for: Temporal interference stimulation for treating Wilson disease: A prospective clinical and functional MRI study
Source: Neurotherapeutics. 2026 Oct 12;23(6):e01097. doi: 10.1016/j.neurot.2026.e01097 (PMC13682462; doi:10.1016/j.neurot.2026.e01097)
Supplement: Supplementary file 1 [file mmc1.docx]

**Supplementary material**

**Title**

Temporal interference stimulation for treating Wilson disease: a prospective clinical and functional MRI study

**Authors**

Nan Cheng, et al.

**Supplementary Table S1.** Individualized transcranial temporal-interference stimulation montage, current allocation, and simulated target electric field for each participant.

**Supplementary Table S2.** MRI data availability and quality-control disposition.

**Supplementary Table S3.** Adverse effects reported following temporal interference stimulation.

**Supplementary Figure S1.** Study design and representative electric-field distribution of right putamen-targeted temporal interference stimulation.

**Supplementary References**

**Supplementary Table S1.** Individualized transcranial temporal-interference stimulation montage, current allocation, and simulated target electric field for each participant.

| **ID** | **Electrode placement** | | **Current (mA)** | | **Simulated target E-field (V/m)** |
| --- | --- | --- | --- | --- | --- |
|  | **I1** | **I2** | **I1** | **I2** |  |
| 1 | PO9-FC2 | F8-F7 | 2.500 | 2.176 | 0.518 |
| 2 | P7-PO10 | AF4-FPZ | 1.922 | 2.140 | 0.216 |
| 3 | PO9-T8 | F7-FCZ | 1.743 | 2.500 | 0.358 |
| 4 | F2-FT9 | CP6-POZ | 1.667 | 2.500 | 0.352 |
| 5 | F6-FC5 | O9-C4 | 2.084 | 2.211 | 0.537 |
| 6 | FT7-AF8 | C6-CP1 | 2.500 | 2.405 | 0.512 |
| 7 | C4-PO9 | FT7-F2 | 1.610 | 2.470 | 0.448 |
| 8 | CP6-PO8 | AF3-F2 | 2.500 | 2.310 | 0.336 |
| 9 | TP7-CP6 | F8-FP2 | 1.129 | 2.500 | 0.286 |
| 10 | AFZ-F4 | TP7-PO10 | 2.412 | 2.500 | 0.337 |
| 11 | FP2-AF3 | CP6-POZ | 2.500 | 2.125 | 0.280 |
| 12 | F3-F2 | C6-TP8 | 1.770 | 2.500 | 0.222 |
| 13 | T8-P10 | F2-F1 | 1.473 | 2.500 | 0.310 |
| 14 | FC2-CP1 | FT8-F10 | 1.357 | 1.964 | 0.403 |
| 15 | AF3-F4 | P5-CP6 | 2.500 | 2.034 | 0.392 |
| 16 | F6-FP2 | O2-PO7 | 1.366 | 2.500 | 0.372 |
| 17 | PO7-F4 | F3-C6 | 2.500 | 1.773 | 0.419 |
| 18 | AF7-F8 | CP4-CP1 | 1.803 | 2.500 | 0.394 |
| 19 | O2-FT8 | TP9-F2 | 1.713 | 2.032 | 0.395 |
| 20 | CP6-TP9 | F8-F3 | 2.215 | 2.065 | 0.481 |
| 21 | F4-F1 | PO9-TP8 | 2.500 | 2.044 | 0.344 |
| 22 | F2-AF3 | TP9-TP8 | 2.500 | 1.555 | 0.309 |
| 23 | C4-PO8 | FT9-FPZ | 1.827 | 2.500 | 0.338 |
| 24 | O1-C6 | FP1-F2 | 1.899 | 2.500 | 0.429 |
| 25 | P3-CP4 | F10-F7 | 2.500 | 2.260 | 0.384 |

**Supplementary Table S2.** MRI data availability and quality-control disposition.

| **Variable** | **Imaging analysis (n=9)** | **Not included (n=16)** | ***P* value** | **SMD** |
| --- | --- | --- | --- | --- |
| Age, years | 30.0 ± 8.9 | 29.8 ± 8.6 | 0.958 | 0.023 |
| Sex, male, n (%) | 7 (77.8%) | 13 (81.3%) | 1.000 | −0.082 |
| BMI, kg/m² | 21.1 ± 3.5 | 21.3 ± 3.0 | 0.900 | −0.054 |
| UWDRS total score | 60.8 ± 42.0 | 58.6 ± 31.2 | 0.888 | 0.064 |
| Neurological | 48.2 ± 37.5 | 46.7 ± 29.8 | 0.916 | 0.044 |
| Hepatic | 4.3 ± 2.5 | 4.2 ± 2.1 | 0.924 | 0.043 |
| Psychiatric | 8.2 ± 4.8 | 7.8 ± 4.7 | 0.839 | 0.084 |

Participants were not included in the imaging analysis due to missing post-treatment MRI data or failure of head motion correction. Continuous variables are presented as mean ± standard deviation, and categorical variables are presented as number (%). P values were calculated using the independent-samples t test or Fisher’s exact test, as appropriate. Standardized mean differences (SMDs) were calculated to assess baseline balance between groups. An absolute SMD value <0.1 was considered indicative of adequate baseline balance.

**Supplementary Table S3.** Adverse effects reported following temporal interference stimulation.

| **Adverse effect** | **TIS group (n=25)** | **Severity** | **Management** | **Outcome** |
| --- | --- | --- | --- | --- |
| Scalp tingling sensation | 1 (4.0%) | Mild | Brief rest | Resolved |
| Fatigue | 1 (4.0%) | Mild | Supine rest for 30 min | Resolved |
| Headache | 0 | Any | - | - |
| Burning sensation | 0 | Any | - | - |
| Warmth sensation | 0 | Any | - | - |
| Dizziness | 0 | Any | - | - |
| Nausea | 0 | Any | - | - |
| Metallic taste | 0 | Any | - | - |
| Phosphene (white light) | 0 | Any | - | - |
| Other sensations | 0 | Any | - | - |

Data are presented as n (%). Severity was graded according to a five-point scale (0=none, 1=mild, 2=moderate, 3=severe, 4=very severe).

**Supplementary Figure S1.** Study design and representative electric-field distribution of right putamen-targeted temporal interference stimulation. (a) Schematic overview of the study procedure, including baseline clinical and MRI assessment, MRI-based individualized right putamen-targeted TIS delivered over 10 sessions across 2 weeks, and post-treatment clinical and MRI assessment. (b) Representative simulated electric-field distribution of the optimized stimulation montage in coronal and axial views, with the predefined right putamen target indicated by the crosshair. The color scale represents electric-field magnitude in V/m.

**Reference**

1. Schaeffer EL, Haber I, Fan Z, et al. Enhancement of sleep slow wave activity using transcranial electrical stimulation with temporal interference: an interim analysis of the STRENGTHEN study. Commun Med (Lond). Published online July 1, 2026.
2. Savvateev I, Missey F, Beliaeva V, et al. Multipair phase-modulated temporal interference electrical stimulation combined with fMRI. Cell Syst. 2026;17(7):101610.
